# Conventional CARs versus modular CARs

**DOI:** 10.1007/s00262-019-02399-5

**Published:** 2019-09-21

**Authors:** Anja Feldmann, Claudia Arndt, Stefanie Koristka, Nicole Berndt, Ralf Bergmann, Michael P. Bachmann

**Affiliations:** 1 Helmholtz-Zentrum Dresden-Rossendorf (HZDR), Institute of Radiopharmaceutical Cancer Research, Bautzner Landstraße 400, 01328 Dresden, Germany; 2 German Cancer Consortium (DKTK), partner site Dresden and German Cancer Research Center (DKFZ), Heidelberg, Germany; 3 University Cancer Center (UCC) Dresden, Tumor Immunology, Carl Gustav Carus’ Technische Universität Dresden, Dresden, Germany; 4 National Center for Tumor Diseases (NCT), Partner Site Dresden, Dresden, Germany

**Keywords:** Immunotherapy, Chimeric antigen receptor, T cells, UniCAR, BiTE, TIMO XIV

## Abstract

The clinical application of immune effector cells genetically modified to express chimeric antigen receptors (CARs) has shown impressive results including complete remissions of certain malignant hematological diseases. However, their application can also cause severe side effects such as cytokine release syndrome (CRS) or tumor lysis syndrome (TLS). One limitation of currently applied CAR T cells is their lack of regulation. Especially, an emergency shutdown of CAR T cells in case of life-threatening side effects is missing. Moreover, targeting of tumor-associated antigens (TAAs) that are not only expressed on tumor cells but also on vital tissues requires the possibility of a switch allowing to repeatedly turn the activity of CAR T cells on and off. Here we summarize the development of a modular CAR variant termed universal CAR (UniCAR) system that promises to overcome these limitations of conventional CARs.

## Introduction

**Fig. 1:**
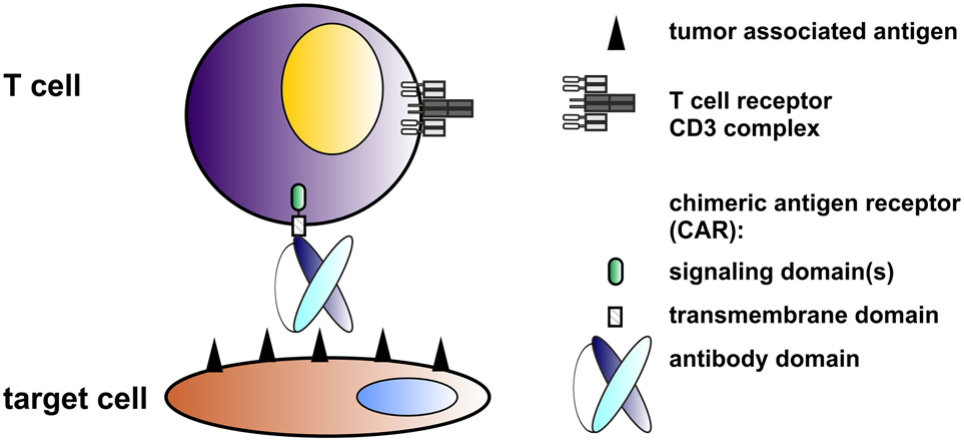
Construction of conventional CARs. A conventional CAR consists of an Ab-based extracellular domain, a transmembrane domain and intracellular signaling domains. The extracellular domain is directed against the tumor-associated antigen (TAA) on the surface of the target cell. It can be constructed from the variable domains of the heavy and light chains of a mAb. After transduction of the T cell with the CAR gene, the resulting CAR T cell can recognize tumor cells via its extracellular Ab domain. Cross-linkage of the tumor cell with a CAR T cell leads to the formation of a synapse-like structure by clustering of the CAR receptors and thereby to an activation of the CAR T cell via its signaling domain(s) and finally to the destruction of the target cell. Both CD4- and CD8-positive T cells work equally well as killer cells though CD4-positive T cells need longer time for cytotoxic activity [29]

**Fig. 2:**
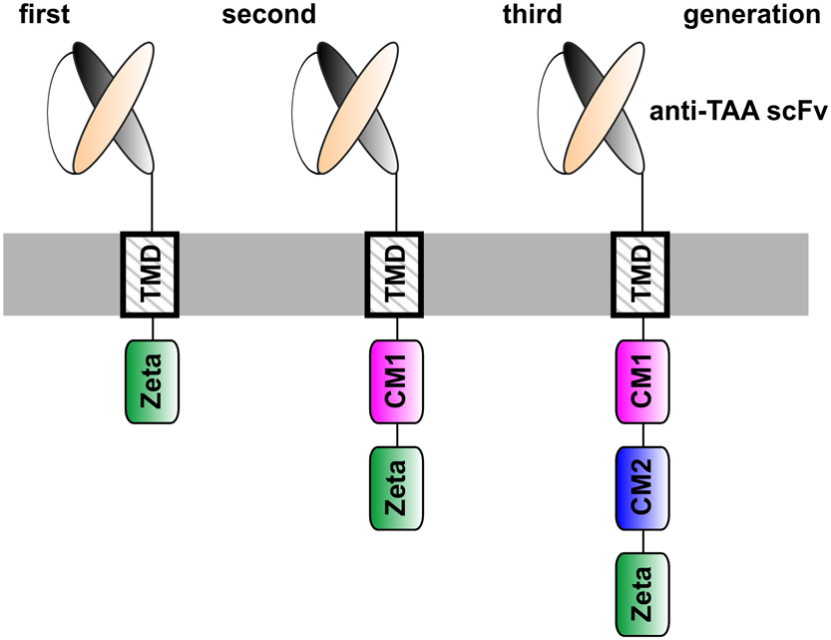
Different CAR concepts. The activation domain in first-generation CARs is taken from the zeta chain of the CD3 complex. To improve survival and reactivity of CAR T cells, second- and third-generation CARs contain, in addition to the activation motif, one or two costimulatory motifs. *CM* costimulatory motif, *TAA* tumor-associated antigen, *TMD* transmembrane domain

After almost 3 decades of development, in which renowned scientists around the world were involved, T cells genetically modified to express artificial receptors (termed as chimeric antigen receptors, CARs) have finally arrived in the clinic [for reviews, e.g., 1–3]. Moreover, two anti-CD19 CARs (Tisagenlecleucel, Axicabtagene ciloleucel) were approved by the U. S. Food and Drug Administration (FDA) for the treatment of relapsed/refractory B-cell acute lymphoblastic leukemia (B-ALL) and diffuse large B-cell lymphoma (DLBCL) [4–6]. Proof of concept for the underlying original idea was already published at the end of the 1980s by the group around Z. Eshhar [e.g., 7, 8]. CARs consist of an extracellular target recognition domain, a transmembrane domain (TMD) and intracellular signaling domain(s) [e.g., 9–11, see also Fig. 1]. The extracellular domain of a CAR is commonly constructed from the variable domains of the heavy and light chains of a monoclonal antibody (mAb) in a single-chain fragment variable (scFv) format. Signaling domain CARs usually contain the CD3ζ chain. However, other activating receptors such as the DAP12 chain work equally well in both NK and T cells [12, Bachmann unpublished]. The activation motif can be combined with one or more costimulatory motif(s) (CM(s)). CMs are commonly taken from CD28, 4-1BB (CD137), ICOS or OX40 (CD134) [e.g., 10, 11, 13, 14]. CARs containing only the CD3ζ chain are known as first-generation CARs (see also Fig. 2). To improve survival and reactivity of CAR T cells, second- and third-generation CARs contain either one or two additional CMs. For gated targeting strategies, the signaling and costimulatory domains can be separated into two CARs [e.g., 15]. This review summarizes the concept and success of CAR T-cell therapy as well as its remaining challenges and potential solutions. Compared to our previously published reviews [16, 17], this one focuses on the development of two switchable universal CAR (UniCAR) systems as an improvement of conventional CAR T-cell therapy.

### Remaining challenges

Although their clinical efficacy is impressive, current CAR T cells are far from being a safe drug. After adoptive transfer, CAR T cells are confronted to numerous tumor cells expressing their target. As a consequence, the CAR T cells will be activated, proliferate and produce tremendous amounts of proinflammatory cytokines which can lead to serious CRS. In addition, the rapid disintegration of a huge amount of tumor cells can cause TLS. Furthermore, during anti-CD19 CAR T-cell treatments, deadly edema occurred in the central nervous system. In case the respective tumor cell target is not only overexpressed on tumor tissues but is also accessible on the surface of healthy tissues, CAR T cells will attack these healthy tissues as well which can cause life-threatening on-target/off-tumor effects [e.g., 18]. On-target/off-tumor effects can even occur during an anti-CD19 CAR T-cell therapy as CD19 is also expressed on the surface of healthy B cells. Fortunately, the loss of B cells is not life-threatening as the lack of antibodies (Abs) can be substituted. However, such a rescue is more or less unique for the target CD19 and not possible in case tumor-associated antigens (TAAs) are targeted that are accessible on the surface of vital tissues. To increase the safety of CAR T cells, a series of strategies has already been described including, e.g., the use of suicide genes, the introduction of an apoptosis switch with the CRISPR/Cas9 system, the application of inhibitory CARs, or the targeting of co-expressed surface antigens, e.g., a truncated version of epidermal growth factor receptor (EGFR) with Cetuximab [19–23]. The obvious task of all of these safeguards is the elimination of the CAR T cells. However, can such strategies work fast enough for the treatment of acute CRS or TLS or central nervous system problems? Most probably not; as recently summarized, full-size Abs or related recombinant derivatives require up to 48 h to be enriched in a solid tumor as is known from positron-emission tomography (PET) imaging [16, 17]. Even 2 h after application, the majority of mAb is still in the peripheral blood and only little is found in the tumor. Thus, a rapid steering with full-size mAbs appears quite unlikely. Similar time problems may occur when a genetic approach should be applied to destroy CAR T cells.

Nonetheless, all these approaches may be useful to protect healthy tissues that express cross-reactive epitopes against the attack of CAR T cells; if applied once, all tumor cells are destroyed. Indeed, anti-CD19 CAR T cells could be eliminated experimentally in mice [23], although it still might remain challenging to find the right time point for patients; obviously, if applied too late, on-target/off-tumor effects will occur. If eliminated too early, remaining tumor cells may lead to recurrent disease. Unfortunately, the sensitivity of currently available imaging technologies is not sufficient to detect few remaining tumor cells. Even in case improved imaging tools may become available, one still would not know about the sensitivity of the detectable tumor cells against the CAR T cells. It could easily be that the remaining tumor cells have downregulated the target recognized by the CAR T cells. For all these reasons, it would be much better not to eliminate the CAR T cells but to regulate their function, e.g., to equip CAR T cells with a rapid and reliable switch to adapt their activity and specificity in case tumor escape variants occur, and to target more than one TAA to reduce the risk for escape variants.

### Modular CARs as solution

In 2012, Urbanska et al. described a modular artificial receptor approach. Instead of an anti-TAA Ab domain, the authors used chicken avidin as the extracellular receptor domain of the artificial receptor [24]. T cells modified with such an artificial avidin receptor were able to target tumor cells via biotinylated adaptor molecules, e.g., biotinylated Abs. However, chicken avidin (or bacterial streptavidin) might be highly immunogenic in humans. Furthermore, the presence of natural anti-biotin Abs in sera of healthy individuals might limit the use of avidin-based receptors in humans [25]. Bearing in mind that full-size Abs have a half-life of several weeks and require at least 24–48 h to enrich at the tumor site, one can expect that adaptor CARs armed with Abs behave more or less like conventional CARs with little chance of a rapid regulation. For these reasons, a rapid switch off, e.g., for the treatment of CRS might not work at least not with adaptor molecules based on full-size Abs. Moreover, if the target is also expressed on the cell surface of blood or endothelial cells, intravenously applied adaptor/CAR T-cell complexes might first attack these healthy cells rather than to leave the bloodstream to find the tumor cells.

So how to solve these problems? While we tried to establish novel bispecific Abs (bsAbs) [e.g., 26–31], we learned that even minor changes in one of the two Ab domains did not only affect the altered Ab domain but also the non-modified one. To easily compare different anti-CD3 and anti-TAA domains, we created a modular bsAb format. For this purpose, we split the bsAb into two components: (1) an effector molecule (EM) and (2) a target molecule (TM). The original idea was to create a bsAb that can be used as a universal EM: for that purpose, on the one hand, the EM is directed to CD3 and, on the other hand, to a suitable peptide epitope. TMs were designed to consist of an anti-TAA scFv fused to the peptide epitope [e.g., 32–34]. Thus, EM and TM can form an immune complex. For proof of concept, we tested a series of peptide epitope tags including, e.g., the oligo(Histidine)-tag or the Myc-tag which were not functional. For several reasons including a low risk of immunogenicity we finally selected two peptide sequences termed E5B9 and E7B6. Both epitope sequences derive from the human nuclear autoantigen La also known as Sjögren’s syndrome-associated antigen B (SS-B) (for more details, see below and also [35]). Interestingly, we found that the respective EM/TM complexes can functionally replace conventional bsAbs [32–34]. This modular bispecific T-cell engager (BiTE) format was termed universal, modular BiTE system (UniMAB).

**Fig. 3:**
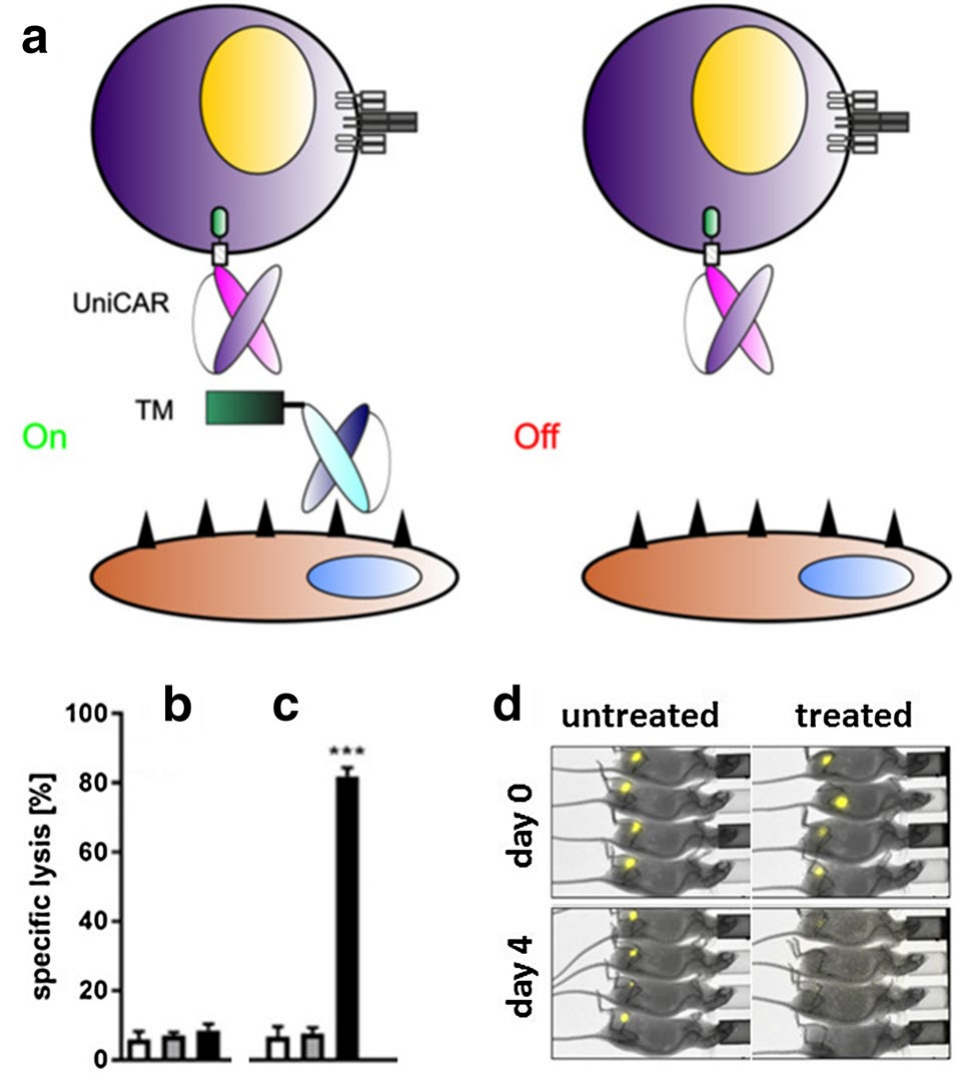
The UniCAR system, a modular switchable CAR platform. **a** Based on anti-La mAbs (either 5B9 or 7B6 directed towards the amino acid (aa) sequence (S)KPLPEVTDEY (UniCAR epitope, note the N-terminal serine (S) is not necessarily required for immune reactivity of the 5B9 mAb but is commonly present in E5B9-containing UniCAR TMs due to G_4_S linkers between the scFv and the UniCAR epitope) or the aa EKEALKKIIEDQQESLNK we constructed the respective UniCAR domain. After adoptive transfer, UniCAR T cells remain in an “Off” mode in the absence of a TM until a TM is infused, which can form a cross-linkage between a UniCAR T cell and the target cell. Consequently, UniCAR T cells can be switched “On” in the presence of a TM and will automatically shut “Off” after elimination of the TM. Prerequisite for a rapid shut down is a short half-life of the TM. An example of selected experimental data for an anti-E5B9 UniCAR system is shown in **b**–**d**. **b** T cells transduced with a non-functional UniCAR (a UniCAR lacking intracellular signaling domains) do not kill target cells neither in the absence (gray bar) nor presence of a TM (black bar). **c** T cells transduced with a signaling UniCAR also do not attack target cells in the absence of a TM (gray bar). However, lysis occurs in the presence of a TM (black bar). **b**, **c** Untransduced T cells (white bars) also fail to destroy target cells. **d** Tumor cells transduced with luciferase can be detected in untreated and treated mice at day 0 by optical imaging. In the absence of a TM (untreated mice), the tumor cells can still be detected 4 days after co-injection of signaling UniCAR T cells. However, no more tumor cells can be detected in treated mice (tumor cells were co-injected with UniCAR T cells in the presence of a TM). Lysis assays and optical imaging were performed as described previously [44]. *TM* target molecule, *UniCAR* universal chimeric antigen receptor

Next, we created CARs based on the same two anti-La epitope scFvs [e.g., 35]. The resulting modular CAR systems were termed UniCARs. Interestingly, all available TMs originally developed for the UniMAB system were also functional in combination with UniCAR T cells. As schematically summarized in Fig. 3, UniCAR T cells (and also the EMs of the respective UniMAB system) are inactive in the absence of a TM (Fig. 3a, Off). After cross-linkage with tumor cells via a TM, however, UniCAR T cells become active (Fig. 3a, On). Therefore, UniCAR T cells can easily be regulated: they can repeatedly be turned “on” just by infusion of the TM and turned “off” by stopping the infusion followed by elimination of the TM. Therefore, most important for a rapid steering of the UniCAR system is that (1) the TM can rapidly reach the target (e.g., by diffusion from the blood to the tumor), (2) form a complex with the UniCAR that can rapidly dissociate (high Off rate), and (3) the TM can rapidly be eliminated from the blood stream. According to PET analysis in experimental mice TMs based on scFvs or nanobodies fulfill these prerequisites [e.g., 36–43]. Such TMs usually have elimination half-lives between 15 and 45 min.

Experimental data for an anti-E5B9-based UniCAR system are presented in Fig. 3b–d. As shown, UniCAR T cells are able to specifically lyse target cells both in vitro (Fig. 3b, c) and in experimental mice (Fig. 3d). Tumor cell killing depends on functional UniCARs and on the presence of a TM: T cells transduced with UniCARs lacking a signaling domain are not able to kill tumor cells (Fig. 3,b), neither in the absence (Fig. 3b, gray bar) nor presence of a TM (Fig. 3b, black bar). Similarly, UniCAR T cells containing a signaling domain cannot kill tumor cells in the absence of a TM (Fig. 3c, gray bar). However, in the presence of a TM, UniCAR T cells containing the signaling domain are able to kill tumor cells (Fig. 3c, black bar).

For first clinical phase 1 trials, we have selected the anti-E5B9 UniCAR version. Between anti-E5B9 scFv and TMD, the extracellular domain of the anti-E5B9 CAR contains the E7B6 epitope sequence as spacer. This E7B6 tag can be used for detection and elimination of the anti-E5B9 UniCARs via anti-E7B6-directed UniCARs if it becomes necessary [35]. In contrast to all other current approaches to destroy unwanted CAR T cells such a procedure would not just eliminate but in parallel replace the anti-E5B9 UniCAR system with the alternative anti-E7B6 UniCAR system.

The E5B9 UniCAR system was first presented at the American Society of Hematology (ASH) meeting in 2014 [36] and already summarized in detail [17]. Since then, other related switchable CAR strategies (e.g., sCARs) were published [44–46].

An obvious problem of such a treatment strategy is: to achieve a sufficiently high concentration of the TM in the patient, it must be applied by continuous infusion. As the treatment may take weeks such a regimen appears inconvenient for patients. However, one could apply a TM with a short half-life at the beginning of a UniCAR therapy, when the tumor burden is high and thus the risk of CRS and TLS might also be high. Once the majority of the tumor is destroyed and these risks are low, one could switch to the application of a TM with a longer half-life. As estimated by PET imaging, immunoglobulin G4 (IgG4)-based TMs have a kinetic comparable to full-size Abs, consequently, TMs based on an IgG backbone are promising candidates for such an application [17]. Until now, we have developed a series of TMs both with short and extended half-lives including, e.g., against CD19, CD123, CD33, prostate stem cell antigen (PSCA), prostate-specific membrane antigen (PSMA), disialoganglioside 2 (GD2), EGFR, cell surface-associated mucin 1 (MUC1), sialyl-Tn (STn), and others [36-43, Bachmann, unpublished]. From these studies we know that TMs can be constructed in a variety of formats. They can be cloned as scFvs from the variable heavy- and light-chain domains of murine or humanized mAbs but also from camelid Abs, so-called nanobodies. Interestingly, TMs can also be prepared from affibodies and soluble T-cell receptors. Even small molecules can be converted into TMs [47]. For example, we successfully created a TM against PSMA by fusion of a UniCAR epitope to a commonly used PSMA PET tracer. The resulting theranostic TM can be used for both retargeting of UniCAR T cells and PET imaging to follow the CAR T-cell therapy in an individualized manner including in humans [47]. In addition to monovalent TMs, we also created bivalent or bispecific TMs. To reduce the risk of tumor escape variants different monospecific TMs or bispecific TMs can be applied either simultaneously or subsequently for (OR) gated targeting [15].

As mentioned above, both UniCAR epitopes were taken from the nuclear autoantigen La/SS-B [48–51]. Both epitopes are cryptic in native La protein as the respective mAb does not coprecipitate native La protein, while synthetic peptides or epitope fusion proteins or denatured La protein reacts with the respective anti-La mAb. The E5B9 epitope consists of the amino acid (aa) sequence KPLPEVTDEY which represents the aa95–104 of the La protein. In the La protein, this peptide sequence is located between the N-terminal La motif and the first ribonucleoprotein consensus sequence. The E7B6 epitope consists of the aa EKEALKKIIEDQQESLNK which represents the aa311–328 of La protein. In native La protein, it forms a helical structure which is located in the C-terminal domain of La protein.

The major reason why we selected UniCARs based on the E5B9 epitope for first phase 1 clinical studies is: over the past decades, we and many other groups have analyzed the structure, function, expression, and also the immune response against the La antigen including in experimental mice and autoimmune patients [e.g., 48–53]. In none of these studies, we saw an immune response against the selected La epitopes in anti-La-positive patients neither at the B nor T cell level. Consequently, even autoimmune patients being able to break tolerance against the La antigen do not develop anti-La Abs against the UniCAR epitope(s). Therefore, it appears rather unlikely that people being tolerant to the nuclear La protein will develop an immune response against the selected La epitopes. But even in the worst case that we would induce an autoimmune response against the UniCAR epitope, anti-La Abs have been reported to be protective against anti-DNA Abs in lupus patients [54]. Another advantage of the selected UniCAR epitopes is that the primary sequence of La protein is conserved during evolution including in rodents [50], so the toxicology of UniCAR T cells can easily be studied in mice.

In summary, the UniCAR system may help to overcome safety issues of the current CAR technology especially in solid tumors.

## Abbreviations

aa: Amino acid
Ab(s): Antibody(ies)
ASH: American Society of Hematology
B-ALL: B-cell acute lymphoblastic leukemia
BiTE: Bispecific T-cell engager
bsAb(s): Bispecific antibody(ies)
CAR(s): Chimeric antigen receptor(s)
CD: Cluster of differentiation
CM(s): Costimulatory motif(s)
CRS: Cytokine release syndrome
DLBCL: Diffuse large B-cell lymphoma
EGFR: Epidermal growth factor receptor
EM(s): Effector molecule(s)
FDA: U. S. Food and Drug Administration
GD2: Disialoganglioside 2
Ig: Immunoglobulin
mAb(s): Monoclonal antibody(ies)
MUC1: Cell surface-associated mucin 1
OMRF: Oklahoma Medical Research Foundation
PET: Positron-emission tomography
PSCA: Prostate stem cell antigen
PSMA: Prostate-specific membrane antigen
scFv: Single-chain fragment variable
SS: Sjögren syndrome
STn: Sialyl-Tn
TAA(s): Tumor-associated antigen(s)
TCR(s): T-cell receptor(s)
TLS: Tumor lysis syndrome
TM(s): Target molecule(s)
TMD: Transmembrane domain
UniCAR: Universal CAR
UniMAB: Universal, modular BiTE
